# The Influence and Role of Microbial Factors in Autoimmune Kidney Diseases: A Systematic Review

**DOI:** 10.1155/2015/858027

**Published:** 2015-05-18

**Authors:** Andreas Kronbichler, Julia Kerschbaum, Gert Mayer

**Affiliations:** Department of Internal Medicine IV (Nephrology and Hypertension), Medical University Innsbruck, Anichstraße 35, 6020 Innsbruck, Austria

## Abstract

A better understanding of the pathophysiology of autoimmune disorders is desired to allow tailored interventions. Despite increased scientific interest a direct pathogenic factor in autoimmune renal disease has been described only in a minority like membranous nephropathy or ANCA-associated vasculitis. Nonetheless the initial step leading to the formation of these antibodies is still obscure. In this review we will focus on the possible role of microbial factors in this context. *Staphylococcus aureus* may be a direct pathogenetic factor in granulomatosis with polyangiitis (GPA). Chronic bacterial colonization or chronic infections of the upper respiratory tract have been proposed as trigger of IgA vasculitis and IgA nephropathy. Interventions to remove major lymphoid organs, such as tonsillectomy, have shown conflicting results but may be an option in IgA vasculitis. Interestingly no clear clinical benefit despite similar local colonization with bacterial strains has been detected in patients with IgA nephropathy. In systemic lupus erythematosus injection of bacterial lipopolysaccharide induced progressive lupus nephritis in mouse models. The aim of this review is to discuss and summarize the knowledge of microbial antigens in autoimmune renal disease. Novel methods may provide insight into the involvement of microbial antigens in the onset, progression, and prognosis of autoimmune kidney disorders.

## 1. Introduction

Over the last decades several investigations have focused on the pathogenesis of immunologically mediated kidney diseases. Besides the detection of novel aspects responsible for the onset of particular diseases, such as the phospholipase A2 (PLA2) receptor [1] and more recently thrombospondin type-1 domain-containing 7A antibodies [2] in membranous nephropathy, there has also been particular interest in the role of microbial agents.

Abnormalities in the immune reactions can induce kidney damage, either by provoking an autoimmune phenomenon or induction of molecular mimicry. The immune response comprises the innate as well as the more orchestrated adaptive immunity. The former acts as the first line of defence and is mediated by monocytes/macrophages, neutrophils, natural killer cells, complement activation, and cytokines. Pathogen-associated small molecules recognized by pattern recognition receptors such as toll-like receptors (TLR), C-type lectin receptors, and complement activation via the mannose-binding lectin pathway initiate innate recognition of bacteria and thus activation of inflammation and coagulation. Once persistent, the antigenic stimulus leads to the activation of the adaptive immunity triggering a T-cell and B-cell response. An autoimmune response occurs whenever the immune system activation leads to a response against self-antigens. Several factors are implicated in the loss of immune tolerance including genetic, hormonal, and environmental factors as well as a defective clearance of apoptotic cells [3–5].

Characterization of the human microbiome [6] offers novel opportunities for a better understanding of these complex phenomena as some commensal bacteria may provoke inflammation while others control it via engagement of TLRs and pathogen-related receptors [7]. Alterations of the microbiota have already been identified in several autoimmune diseases, including inflammatory bowel diseases, type-1 diabetes, and experimental autoimmune encephalomyelitis [8]. However, a negative impact of the microbiota on inflammation and autoimmunity in genetically susceptible subjects has been reported as well. In particular, T-cell modulation as shown by induction of either Th17 or regulatory T-cell responses triggered development or protection from autoimmune/inflammatory disease [7, 9].

Bacterial infection-related glomerulonephritis has attracted attention for a long period of time, for example, with streptococcal infections as leading cause of postinfectious glomerulonephritis in children and staphylococcal infection in adults [10]. In this review, we focus on the microbial influence on the onset and progression of autoimmune kidney disorders.

## 2. Materials and Methods

### 2.1. Search Strategy

A systematic literature search of the MEDLINE database was conducted, using the key words: “(bacteria OR bacter^∗^ OR microbiology OR microbio^∗^ OR microbiome OR microbial OR microorganism OR microbiota) AND (glomerulonephritis OR nephrotic syndrome).” Additional studies were identified by examining the bibliography of the retrieved articles by forward search. The search was limited to articles published in English and reports on postinfectious and IgA-dominant acute staphylococcal associated glomerulonephritis forms were excluded from further analysis.

## 3. Results

### 3.1. Search Results

**Figure 1:**
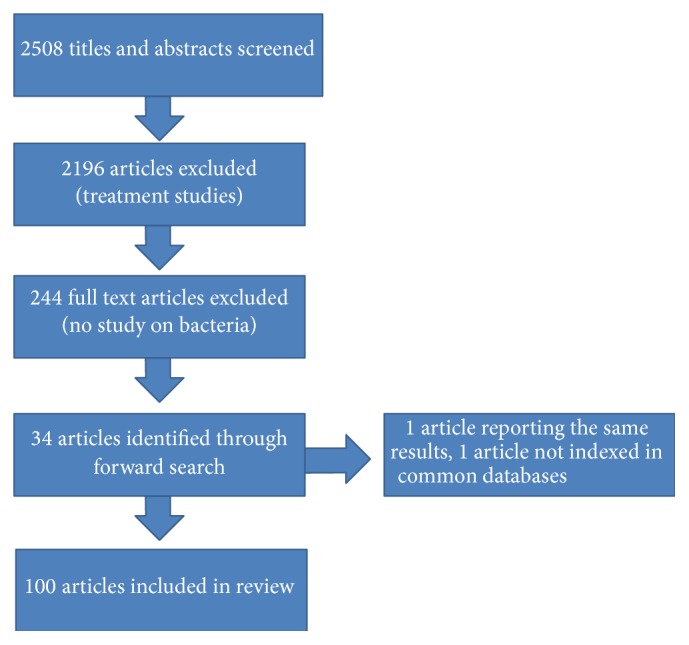
The search strategy is shown. Of initial 2508 titles screened, the majority (2196 articles) were excluded due to not reporting on bacterial influence in primary glomerulonephritis. 244 reports were accessed in full text but could be excluded for not reporting on bacterial agents in primary glomerular diseases (i.e., postinfectious glomerulonephritis or virus-related kidney diseases). The bibliography of the retrieved articles revealed another 34 reports. One record reporting the same results and another one not indexed in common databases were excluded from final analysis, leading to a total number of 100 articles.

The systematic search (performed on July 1, 2014) overall resulted in 2508 records. A large number of articles (*n* = 2196) was excluded, since these records reported on treatment regimens, antibody findings (such as anti-neutrophil cytoplasmic antibodies), and patients with virus-related diseases (such as hepatitis C-associated membranoproliferative glomerulonephritis or human immunodeficiency virus-associated kidney disease) or were single case reports/case series. After access to full text articles we excluded another 244 articles, which were not reporting on bacterial antigens or bacteria leading to autoimmune kidney diseases but rather again reported on poststreptococcal or poststaphylococcal glomerulonephritis or viral antigens, leading to a number of 68 articles for further full text analysis. Through forward search of the retrieved bibliography we identified another 34 records, which were also analyzed. Of these, one article was not indexed in common electronic databases. After exclusion of one article reporting the same results a total of 100 records were finally included (see Figure 1).

### 3.2. Anti-Neutrophil Cytoplasmic Antibody-Associated Vasculitis

The group of anti-neutrophil cytoplasmic antibody- (ANCA-) associated vasculitis (AAV) consists of four distinct diseases, namely, granulomatosis with polyangiitis (GPA, former Wegener's granulomatosis), microscopic polyangiitis (MPA), eosinophilic granulomatosis with polyangiitis (EGPA, former Churg-Strauss syndrome), and renal limited vasculitis. The extent of kidney involvement varies between and within each clinical entity being around 90–100% in MPA, 50–80% in GPA, and, depending on the ANCA status, 4–51% in EGPA [11]. Several genetic traits, environmental factors, and the use of specific medications have been identified as potential triggers to develop AAV.

#### 3.2.1. Granulomatosis with Polyangiitis: A Role for* Staphylococcus aureus*?

Bacterial antigens potentially implicated in the pathogenesis of AAV have been studied for three decades. In a prospective longitudinal cohort the chronic presence of* Staphylococcus aureus* obtained from nasal swab cultures was identified as an independent risk factor for relapse of GPA when compared to noncarriers. Chronic* Staphylococcus aureus* carriers were defined as a positive culture in ≥75% of the specimen (mean of 22.6 cultures per patient, follow-up for at least one year) examined [12]. A subsequent prospective, randomized, placebo-controlled trial with a majority of GPA patients suffering from renal involvement evaluated the efficacy of trimethoprim-sulfamethoxazole twice daily for 24 months and revealed a significant reduction of disease relapses. It was postulated that the elimination or reduction of* Staphylococcus aureus* may be responsible for this beneficial effect, although nasal swabs were not performed. In addition, a significant reduction of respiratory and nonrespiratory tract infections was reported in actively treated patients [13]. Chronic nasal colonization of* Staphylococcus aureus* (positivity in ≥80% of samples obtained) was furthermore confirmed as independent risk factor for relapse in biopsy-proven GPA in another cohort [14]. A retrospective longitudinal cohort study revealed that a high proportion of GPA patients (>70%) carried at least one* Staphylococcus aureus* superantigen, which can lead to an antigen-independent T-cell activation. In general, the presence of* Staphylococcus aureus* was accompanied by a higher risk for relapse, which however was modulated by the type of superantigen present, with mostly tsst-1 being associated with disease relapse [15]. Further investigations to address whether* Staphylococcus aureus* or related superantigens are capable of T-cell expansion were performed. T-cell expansion was present at a significantly higher rate in patients with GPA than in healthy individuals but was neither associated with the presence of* Staphylococcus aureus* nor associated with its superantigens [16]. In order to elucidate further pathogenetic consequences of* Staphylococcus aureus* presence in vasculitis, staphylococcal acid phosphatase (SAcP) and its binding ability were studied in human umbilical vein endothelial cells. SAcP was capable of binding to endothelial cells in a concentration-dependent manner and endothelial cell-bound SAcP was recognized by sera of patients with GPA [17]. Immunization of rats with* Staphylococcus aureus* (*n* = 7) led to the development of pauci-immune glomerulonephritis in one rat [18].

#### 3.2.2. Molecular Mimicry in Pauci-Immune Glomerulonephritis: A Role for Gram Negative Bacteria?

In patients with pauci-immune glomerulonephritis, gp130 was detected in about 90% of patients in their active phase and frequently coappeared with myeloperoxidase and proteinase 3 [19]. Gp 130 is identical with human lysosomal associated membrane protein (hLAMP-2) which again shares a 100% homology of the epitope P_41–49_ to the amino acids 72–80 of mature bacterial adhesin FimH, which is present on the tip of fimbriae crucial for attachment to host epithelia of Gram negative bacteria such as* Escherichia coli*,* Klebsiella pneumonia*, and* Proteus mirabilis*. A retrospective survey revealed a high proportion of Gram negative infections prior to the onset of disease activity and autoantibodies to hLAMP-2 were detected in nearly all patients with renal involvement and underlying AAV in one study. The same authors reported that hLAMP-2 autoantibodies, when injected into rats, were causative of developing focal necrotizing glomerulonephritis and a monoclonal antibody directed against hLAMP-2 induced apoptosis of human microvascular endothelium* in vitro* [20]. However, another group of investigators was not able to confirm these interesting findings when injection of anti-hLAMP-2 antibodies into rats did not provoke onset of glomerulonephritis [21]. Nonetheless* Escherichia coli* immunization of rats led to induction of pauci-immune glomerulonephritis and detectable ANCA in one out of eight animals [18] and another experimental study revealed that bacterial lipopolysaccharide (LPS) dose-dependently increased renal injury induced by anti-MPO directed immunoglobulin G (IgG) as demonstrated by the formation of glomerular crescents [22] (Table 1).

#### 3.2.3. Conclusion

In summary in GPA nasal carriage of* Staphylococcus aureus* seems to play a pivotal role in the disease onset and relapse. As far as Gram negative bacteria are concerned there is some evidence that cross reactivity of antibodies directed against surface adhesin FimH, which has structural homology with hLAMP-2 against endothelial cells, can cause vasculitis. However this evidence comes out of one single group and still needs confirmation [19, 20, 23].

### 3.3. IgA Vasculitis

Immunoglobulin A vasculitis (IgA vasculitis, formerly known as Henoch-Schönlein purpura) is a small vessel vasculitis with predominantly deposition of IgA1 within the capillaries, venules, or arterioles of affected tissues. The disease affects the skin and the gastrointestinal tract in a majority of patients and glomerulonephritis, indistinguishable from IgA nephropathy on kidney biopsy, is present [24] in approximately half of the cases. The risk of developing chronic kidney disease (CKD) is higher in adults when compared to children and varies between 35% and 69% [25].

Evidence for a potential microbial influence on the pathogenesis stems from the observation that onset of vasculitis is frequently preceded by an upper respiratory tract infection. It was proposed that the tonsils play an especially important role and chronic infections of the oropharynx and the middle ear can be detected in a majority of children with IgA vasculitis with apical periodontitis, rhinosinusitis, and otitis media being present in 53%, 48%, and 10%, respectively. In one report eradication of the infectious focus (administration of antibiotics and root canal therapy) resulted in a permanent remission without recurrence of disease in 31 out of 40 patients [26]. Nephritis-associated plasmin receptor (NAPI-r), a group A streptococcal antigen, was detected in ten out of 33 patients within the glomerular mesangium of patients with IgA vasculitis, whereas NAPI-r staining was present in only four out of 120 patients with other renal disorders. Moreover, patients with IgA vasculitis showed significantly higher antistreptolysin O (ASLO) titers than patients with other renal diseases and IgA vasculitis patients with positive intraglomerular NAPI-r staining exhibited significant higher ASLO titers than those without [27]. In another prospective study ASLO titer positivity was associated with a 10-fold increase in the risk of developing IgA vasculitis [28] and examination of kidney biopsies revealed presence of IgA-binding region of three different streptococcal M proteins, namely, M4, M22, and M60, in seven out of 13 subjects. On electron microscopy these were present within the glomerular basement membrane (GBM) and the mesangial matrix. Analysis of skin biopsies obtained from IgA vasculitis patients indicated the presence of the same M proteins in the majority of patients investigated but these were absent in kidney biopsy specimens of patients with anti-GBM disease, postinfectious glomerulonephritis, lupus nephritis, and membranous nephropathy [29]. Additionally, a role of* Staphylococcus aureus* has been proposed in IgA vasculitis when it was shown that the percentage of T-cell receptor- (TCR-) V*β*-(5.2 + 5.3) and V*β* 8-positive cells, members of the T-cell repertoire with marked expression after stimulation with staphylococcal enterotoxin, was significantly increased in patients with active disease when compared to healthy individuals whereas specific TCR-V*β* was not present when infection was controlled [30].

Additional microbes have been alleged to contribute to the pathogenesis of IgA vasculitis. Immunohistochemistry of kidney specimen revealed presence of* Helicobacter pylori* antigen in 100% of samples investigated and the positive staining was restricted to glomerular structures [31]. Mesangial staining against* Haemophilus parainfluenzae* antigen was detected in 35% of kidney biopsy samples but only in 4% in other kidney diseases. Moreover, significantly higher plasma IgA1 antibody levels against* Haemophilus parainfluenzae* were present in IgA vasculitis than in patients with other renal diseases [32] (Table 2).

#### 3.3.1. Conclusion

In summary there might be a role of bacterial infections in IgA vasculitis with the tonsils as a mediator of ongoing inflammation. Streptococcal M proteins along with NAPI-r can be detected in renal biopsies, while ASLO titers were significantly elevated when compared to other renal disorders [27–29]. Other papers revealed presence of* Helicobacter pylori* and* Haemophilus influenzae* in respective kidney biopsies, while IgA1 antibodies were more abundantly found in the latter case than in patients with other kidney diseases [30, 31]. IgA vasculitis associated with* Staphylococcus aureus* infections has also been described [30]. Tonsillectomy as a therapeutic option yielded excellent remission rates in refractory IgA vasculitis patients in small cohorts, further supporting a critical role of the tonsils in the pathogenesis [33].

### 3.4. IgA Nephropathy

IgA nephropathy is the most common glomerulonephritis and is characterized by presence of mesangial deposition of IgA1 subclass, which is deficient in galactose. A majority of children or adolescents affected present with macroscopic hematuria (AE) shortly after an upper respiratory tract or gastrointestinal infection, whereas in contrast adult patients commonly present with microscopic hematuria, proteinuria, and hypertension. Microbes have been implicated in etiopathogenesis for a long time as it was assumed that an infection may facilitate synthesis of anti-glycan antibodies cross-reacting with galactose-deficient IgA1. For the nephritogenicity of galactose-deficient IgA1 formation of immune complexes is critical [34].

#### 3.4.1. Bacteria Implicated in IgA Nephropathy Compared to IgA Vasculitis

In line with this idea, a pivotal role of the tonsils has been proposed. Bacterial strains in patients with chronic tonsillitis and IgA nephropathy showed a similar distribution and alpha streptococci were most abundantly present in both groups [35]. When compared to IgA vasculitis periodontal disease and rhinosinusitis (55% and 18% of patients) were present at a similar frequency in patients with IgA nephropathy, whereas otitis media was only seen in the systemic disease [26].

#### 3.4.2. Bacteria Implicated in IgA Nephropathy Compared to Controls

Analysis of tonsil specimens obtained from 68 patients with IgA nephropathy revealed presence of periodontal bacteria like* Haemophilus segnis* and* Campylobacter rectus* in about half of the cases examined, significantly more frequent when compared to controls with chronic tonsillitis, while* Treponema* sp. could be detected in 24% of patients with IgA nephropathy and in 7% of controls (no significant difference). Remission of proteinuria in IgA nephropathy subjects was achieved more often when* Campylobacter rectus* and* Treponema* sp. were present [36]. Examination of 32 patients revealed* Helicobacter pylori* in their palatine tonsils and half of them had coexistence with* Actinomyces israelii* even though the latter finding did not differ for patients with recurrent pharyngotonsillitis only [37]. In another study, tonsillectomy specimens from 14 patients with IgA nephropathy revealed presence of coccoid form* Helicobacter pylori* in all samples and cytotoxin-associated antigen A (CagA) was produced by a majority of the strains [38]. In both studies the authors noted a higher prevalence of* Helicobacter pylori* in palatine glands in patients with IgA nephropathy compared to those with recurrent pharyngotonsillitis only [37, 38].

#### 3.4.3. *In Vivo* Alterations Associated with Microbial Factors in IgA Nephropathy

Blood samples of 21 IgA nephropathy patients indicated significantly higher antibody titers to the IgA-binding region (BR) of streptococcal M proteins M4 and M60 when compared to healthy controls [39]. Recognition of outer membrane of* Haemophilus parainfluenzae* antigens (OMHP) by sera obtained from patients with IgA nephropathy revealed homology with amino acid sequences from outer membrane protein P6 precursor, P5, and P2 porin protein of* Haemophilus influenzae* [40].

Analysis of 35 patients with IgA nephropathy showed presence of IgA antibodies against four of five investigated pneumococcal polysaccharides (7F, 9N, and 14 and 23F), the titers of which were significantly increased when compared to patients with other glomerulopathies, whereas IgG antibodies did not differ between both groups [41]. Moreover, IgA-BR of streptococcal M proteins M4, M22, and M60 could be detected in 10 out of 16 kidney biopsies obtained from patients with IgA nephropathy. These proteins showed the same mesangial and glomerular deposition as observed in IgA vasculitis and colocalization with IgA deposits [29].

Moreover, patients with IgA nephropathy and* Helicobacter pylori* infection showed a greater rate of IgA anti-*Helicobacter pylori* seropositivity and a more pronounced IgA and IgG anti-*Helicobacter pylori* response compared to patients without renal disease, the latter predominantly existing of polymeric IgA1, IgG2, and IgG3 in the diseased group [42].

As already pointed out earlier significantly higher levels of IgA and IgG antibodies directed against OMHP have been observed in patients with IgA nephropathy compared to patients with other glomerular diseases [40] and the titers of IgA antibodies against* Haemophilus parainfluenzae* significantly correlated with the degree of glomerular injury especially in patients with episodes of macroscopic hematuria [43].

Reactivity of IgA eluted from kidney tissues against* Haemophilus influenzae* could be demonstrated in three out of 5 kidney biopsies but the same phenomenon was also observed in two out of 6 non-IgA nephropathy renal specimens [44]. Measurement of IgA, IgM antibody titers against* Escherichia coli* and* Haemophilus influenzae* revealed significantly higher values against both bacteria in IgA nephropathy which correlated with total serum IgA and IgM [45].

IgA class antibodies against* Staphylococcus aureus* were also increased in patients with IgA nephropathy compared to healthy controls. Moreover, the avidity of these antibodies against* Staphylococcus aureus* was significantly lower in patients compared to controls [46]. Interestingly, 79 out of 116 renal biopsy specimens revealed presence of a* Staphylococcus aureus* cell envelope antigen in the glomeruli of patients with IgA nephropathy and colocalisation thereof with glomerular deposits of IgA [47].

#### 3.4.4. *In Vitro* Alterations Associated with Microbial Factors in IgA Nephropathy

Streptococcal M5 isolated from peripheral blood mononuclear cells (PBMC) of IgA nephropathy patients increased surface IgA-positive B cells by 1.6-fold and induced transforming growth factor-*β* in PBMC supernatants by 3-fold compared to controls. In addition, the proliferation capacity of lymphocytes was higher when compared to the one observed in patients with nonproliferative glomerulonephritis [48] and tonsillar lymphocytes showed a significant increase in the IgA1/IgA ratio after stimulation with hemolytic streptococci [49]. An immunologic role has also been proposed for* Helicobacter pylori*-associated CagA. Stimulation of a B-cell line with CagA resulted in cell proliferation and dose-dependently increased production of IgA1 and CagA led to the stimulation of underglycosylated IgA1* in vitro* [50].

Lymphocytes obtained from palatine tonsils of patients with IgA nephropathy showed a significantly higher stimulation index to along with a higher level of IgA and IgA1 antibodies against* Haemophilus parainfluenzae* antigens compared to lymphocytes from patients with chronic tonsillitis [51]. These results were corroborated by others [52], suggesting that* Haemophilus parainfluenzae* antigens are capable of stimulating tonsillar T and B cells in patients with IgA nephropathy. Tonsillar mononuclear cells of patients with IgA nephropathy exhibited a higher capability to produce transforming growth factor-*β* and interleukin-10 along with total IgA after stimulation with OMHP antigens than those from patients with chronic tonsillitis* in vitro* [53]. Moreover, it was shown that* Haemophilus parainfluenzae-*specific IgA is produced by* Haemophilus parainfluenzae* and other bacteria and viruses possessing specific CpG motifs in tonsillar mononuclear cells [54]. Stimulation of tonsillar T cells from IgA nephropathy patients with* Haemophilus parainfluenzae* is enhancing the expression of TCR-V*β*-6* in vitro* and further experiments indicated that the TCR-V*β*-6 was used more frequently in tonsillar T cells of patients with IgA nephropathy and the proportion of these cells in peripheral blood decreased significantly following tonsillectomy compared to controls, suggesting a selective expansion of T cells in IgA nephropathy [55].

Administration of specific pneumococcal C-polysaccharide (PnC) led to deposition of IgA-PnC within the GBM and nuclear factor-kappa B transcription factor was activated early and progressively increased in response to glomerular IgA-PnC deposits in a mouse model [56]. Moreover, in the same experimental setting investigating the role of outer membrane antigen components of* Haemophilus parainfluenzae* indicated a direct nephritogenic effect. Mice either ingesting orally or receiving intraperitoneally OMHP antigens develop glomerular IgA deposition and mesangial expansion, similar to the findings in IgA nephropathy [57].

Induction of glomerulonephritis resembling an IgA nephropathy phenotype was observed in Balb/c mice after immunization with* Staphylococcus aureus* antigens [58]. Immunization with a maltose-binding protein and a 20-amino acid peptide derived from* Staphylococcus aureus* was also capable of inducing an IgA nephropathy like phenotype. Furthermore, anti-20-peptide antibodies labelled glomeruli of patients with IgA nephropathy [59] (Table 3).

### 3.5. Conclusion

In summary IgA nephropathy presents the most common form of glomerulonephritis. Microbial factors have been studied within the tonsils and lymphocytes as well as mononuclear cells extracted from tonsillectomy specimens.* Haemophilus segnis*,* Campylobacter rectus*, and* Treponema* sp. were more abundantly present in tonsils of IgA nephropathy patients examined. Since remission of proteinuria was more frequent in patients carrying* Campylobacter rectus* and* Treponema* sp. [36] a pathogenetic role of these strains can be discussed. Antibodies against pneumococcal polysaccharides and detection of M proteins in kidney biopsies reveal a possible role of streptococci in IgA nephropathy [29, 41]. Administration of streptococcal M5 led to increased proliferation capacity of lymphocytes [48], whereas administration of PnC increased IgA-PnC deposits within the kidney [56]. Stimulation of tonsillar lymphocytes with hemolytic (AE) streptococci led to an increase of the IgA1/IgA ratio* in vitro* [49]. Evidence from* in vivo* and* in vitro* investigations thus suggested a role of streptococci in IgA nephropathy.* Helicobacter pylori* was also present more abundantly in the palatine tonsils, and most of the strains isolated produced CagA [37, 38]. CagA stimulation of a B-cell line led to an increased production of hypoglycosylated IgA1, which is implicated in the pathogenesis of IgA nephropathy [50]. Antibodies against OMHP antigens have been observed in IgA nephropathy patients. Moreover, molecular mimicry of these antigens was shown with other membrane proteins of* Haemophilus parainfluenzae* [40]. Further investigations observed a pronounced tonsillar T and B cell response and a lymphocyte response following stimulation with* Haemophilus parainfluenzae* [51, 52]. Injection or ingestion of OMHP antigens led to the onset of kidney disease, resembling the phenotype of IgA nephropathy [57]. Taken together, there might be an important role of* Haemophilus parainfluenzae*, since OMHP antigens are more abundantly expressed and lead to an IgA nephropathy like phenotype in a mouse model. However, as these results come from one research group, one should interpret the findings with caution.* Haemophilus influenzae* could be eluted from kidney biopsies of IgA nephropathy patients and higher antibody levels against* Haemophilus influenzae* and* Escherichia coli* could be detected [44, 45]. More evidence is clearly needed to support a role of both bacterial strains in the pathogenesis of IgA nephropathy. A clear-cut role has been proposed for* Staphylococcus aureus* in IgA nephropathy, since* Staphylococcus aureus* antibodies directed against IgA were increased [46], a* Staphylococcus aureus* envelope antigen was present in glomeruli of a majority of patients [47], and immunization of Balb/c mice with* Staphylococcus aureus* antigens led to a phenotype resembling IgA nephropathy [58]. A therapeutic role of performing tonsillectomy has been proposed for several years. A recent multicenter (AE) study revealed no benefit regarding clinical remission rates, although proteinuria decreased in a significant manner [60].

### 3.6. Recent Advantages and Future Perspectives

A recent genome wide association study revealed novel associations of IgA nephropathy and loci associated with inflammatory bowel disease or maintenance of the intestinal epithelial barrier and potential alteration in the response to mucosal pathogens, further highlighting an interplay between the intestine and the kidneys in IgA nephropathy [61]. These findings are in line with observations that enteric budesonide administered to abate intestinal inflammation significantly reduced proteinuria and improved kidney function in a small preliminary study [62]. Further insights into intestine-kidney interaction may come from studies looking into the effects of enteric budesonide on the intestinal epithelial barrier and changes in the composition of gut microbiota. A recently published study investigated patients with “progressive” versus “nonprogressing” IgA nephropathy and subjects with an impaired kidney function showed a lower diversity of intestinal microbiota [63]. However, further studies are clearly warranted since in particular stool microbiome analysis is still in its infancy and several potential flaws (i.e., contamination of DNA extraction kits) may influence results.

### 3.7. Nephrotic Syndrome

Nephrotic proteinuric renal diseases comprise three distinct entities, membranous nephropathy, focal segmental glomerulosclerosis (FSGS), and minimal change disease (MCD). In idiopathic membranous nephropathy the target antigen has been identified with PLA2 receptor antibody positivity in approximately 70% of patients [1], with the number increasing to over 80% in untreated patients [64]. More recently, in patients with PLA2 receptor antibody negativity, thrombospondin type-1 domain-containing 7A antibodies have been identified as a circulatory factor in 10% of the remaining patients [2]. The pathogenesis of FSGS and MCD has been studied intensively, but convincing pathogenic factors have not been elucidated so far. While membranous nephropathy and focal segmental glomerulosclerosis tend to progress to end stage renal failure in some patients [65, 66], minimal change disease exhibits a more benign disease course [67].

In membranous nephropathy, the infection rate of* Helicobacter pylori* as detected by a high-molecular-weight cell-associated protein test was found to be significantly higher (66%) when compared to an age-matched control group without history of kidney disease (44%). When eradication was achieved in four patients, remission was reported in three of them. However, the disease course may have been influenced by concomitant glucocorticoid treatment as well [68]. Examination of kidney biopsy specimens showed* Helicobacter pylori* antigen in about two-thirds of the patients with membranous nephropathy [31], a number similar to the one described in an earlier study [69].

Conflicting results regarding the frequency of urinary tract infections preceding onset of nephrotic syndrome in children have been reported. While in an Indian cohort urinary tract infections were common in patients with non-steroid responsive nephrotic syndrome or during relapse with approximately 40% [70], only one out of 32 Nigerian children with nephrotic syndrome presented with urinary tract infection at the initial diagnosis [71] (Table 4).

#### 3.7.1. Conclusion

Microbial antigens may play a role in membranous nephropathy as was shown by an abundance of* Helicobacter pylori* antigen deposition in renal biopsy specimens and evidence of infection in blood [31, 68]. Further investigations to clarify the role of infections prior to onset of nephrotic syndrome are clearly warranted, since urinary tract infections are transiently increasing proteinuria and might be a first step in the onset of nephrotic diseases. Bacterial lipopolysaccharide was capable of inducing CD80, a proposed factor involved in the onset of proteinuria [72]. Costimulation blockade with abatacept induced complete or partial remission in five patients with severe to treat FSGS [73]. Thus, molecular mimicry or a direct role of bacterial strains may be at least partially responsible for CD80 upregulation.

### 3.8. Systemic Lupus Erythematosus and Lupus Nephritis

Systemic lupus erythematosus is a remarkable complex autoimmune disorder with considerable heterogeneity in the onset of symptoms, the presentation of organ involvement, and therapeutic response towards immunomodulatory as well as immunosuppressive medication. Renal involvement, present in about 50% of patients within the first year after diagnosis, is one of the most important hallmarks necessitating more intensive immunosuppression. Antibodies directed against nuclear antigens play a pivotal role in the development and monitoring of the disease [74].

Presence of* Helicobacter pylori* in kidney biopsy specimens from lupus nephritis patients (classes I–V) was observed in 18 out of 27 patients in one study [31]. However, other associations of bacterial strains with kidney disease or lupus disease activity have not been shown yet.

More evidence for an impact of microbes in SLE largely derives from animal models and* in vitro* experiments. Cross-reactivity of anti-pneumococcal antibodies obtained from a patient after vaccination has been shown with foreign- and self-antigens. Moreover, these antibodies exerted the capability to bind anti-double-stranded DNA antibodies, indicating a potential role of molecular mimicry [75]. Further investigations revealed that in total eight antibodies either reacting with pneumococcal saccharide or DNA were able to bind glomerular structures. Of these, six bound to renal protein antigens which had previously been described to be cross-reactive with DNA, whereas the remaining two bound to histones [76]. Effects of two additional bacteria could be shown more recently.* Escherichia coli* DNA and CpG-oligodeoxynucleotides increased DNA antibodies in lupus mice, accompanied by progression of mild to crescentic glomerulonephritis, interstitial fibrosis, and heavy proteinuria [77]. Cholera toxin B, a component of* Vibrio cholerae*, promoted autoantibody production and onset of glomerulonephritis in lupus prone mice [78]

Several investigations in mice highlighted that injection of bacterial lipopolysaccharide induced anti-double-stranded DNA antibodies [79–81]. Moreover, it was shown that glomerular pathology worsened during the time of follow-up after injection of lipopolysaccharide with progressive deposition of immune complexes [80]. These findings could further be corroborated by an increased deposition of immune complexes in kidneys and exacerbated lupus nephritis following exposure to lipopolysaccharide [82]. The effects executed by lipopolysaccharide lasted for six weeks and glomerular dysfunction progressed from nephritis to permanent chronic kidney damage [83]. After injection of lipopolysaccharide a shift in lupus nephritis, from mesangial expansion to necrosis of capillary loops, epithelial proliferation, and glomerulosclerosis with concomitant renal insufficiency and increasing proteinuria, has been observed [84] (Table 5).

#### 3.8.1. Conclusion

In systemic lupus erythematosus, at least mouse models indicate a role of bacteria in the development and progression of nephritis, associated with an increase in immune complexes, polyclonal B-cell activation, increasing proteinuria, and irreversible kidney damage. Furthermore,* Helicobacter pylori* antigen could be detected in two-thirds of the kidney biopsies examined [31]. However, a pathogenetic role thereof is doubtful. Further investigations in humans are clearly warranted to corroborate these findings. Since pathogenic steps leading to the onset of systemic lupus erythematosus still remain to be in the dark, studying microbial agents may be one of the fields of interest.

## 4. Discussion and Future Perspective

Bacterial antigens are implicated in the onset and in the progression of many autoimmune kidney disorders. There is evidence that* Staphylococcus aureus* exerts a direct pathogenic role in nasal epithelia in GPA. Other bacteria may not be directly related to the development of autoimmune kidney disorders, but molecular mimicry or deposition of immune complexes secondary to infections may contribute to renal involvement or damage.

However there are several limitations when assessing data about the role of microbial agents in autoimmune kidney disorders. Some of the most interesting findings have been proposed by single research groups, like the findings of hLAMP-2 in pauci-immune crescentic glomerulonephritis or the role of* Haemophilus parainfluenzae* in IgA nephropathy. Nevertheless, both proposed implications rely on robust data. Moreover, some of the investigations have focused on detection of antigens in kidney biopsies or eluting antigens from kidney biopsy samples. There is a strong need to corroborate such associative findings in more convincing pathomechanistic experimental models. The lack of proof of causality is also a problem with studies in which an abundance of bacterial strains was detected in tonsils of patients with IgA nephropathy, as no satisfying effect of tonsillectomy to induce clinical remission was demonstrated in a recent multicenter (AE) trial even though the operation was able to mitigate proteinuria [60].

More investigations with novel methods (i.e., examination of the human microbiome) will yield a way to better define the role of bacteria in these diseases. Several sites of interest such as the sinuses in GPA, the tonsils and the intestine in IgA vasculitis, and IgA nephropathy may show significant alterations in bacterial colonization diversity, which in turn may contribute to an immunologic imbalance (i.e., altered T-cell homeostasis) leading to local inflammation and provoking the onset or the recurrence of disease. Since studies of the human microbiome already offered insights into several other autoimmune disorders, we are convinced that more profound analyses with longitudinal sample collection (onset of disease, remission, and relapse) will also clarify at least in part etiopathogenesis of some autoimmune kidney disorders. Furthermore, results obtained from microbiome analysis may enable us to prescribe tailored therapeutic measures aiming to eliminate abundant strains, such as trimethoprim-sulfamethoxazole treatment in limited GPA or eradication of infectious foci in IgA vasculitis, potentially restoring microbial and epithelial barrier imbalance.

## Figures and Tables

**Table 1:** Study characteristics of relevant studies in ANCA-associated vasculitis are shown. Most reports investigated the role of *Staphylococcus aureus* in GPA, but more recently a role of molecular mimicry with Gram negative bacteria was proposed as investigated by Kain et al. as well as by Peschel et al. [19, 20, 23] and Roth et al. [21].

Reference	Design	Disease	Subjects	Biological agents
Stegeman et al. [12]	Cohort study	GPA	71 humans	*Staphylococcus aureus *

Stegeman et al. [13]	RCT	GPA	81 humans	*Staphylococcus aureus *

Zycinska et al. [14]	Cohort study	GPA	28 humans	*Staphylococcus aureus *

Popa et al. [15]	Cohort study	GPA	62 humans	*Staphylococcus aureus *

Popa et al. [16]	(1) Cross sectional (2) Cohort study	GPA	(1) 36 humans (2) 10 humans	*Staphylococcus aureus *

Brons et al. [17]	Cell study	GPA	Human umbilical vein endothelial cells (HUVECs), glomerular endothelial cells	*Staphylococcus aureus *

Savige et al. [18]	Animal study	AAV	(1) 7 adult male Wistar rats (2) 8 adult male Wistar rats	(1) *Staphylococcus aureus* (2) *Escherichia coli *

Kain et al. [19]	Cross sectional	Pauci-immune glomerulonephritis	16 humans	Molecular mimicry with Gram negative bacteria

Kain et al. [20]	(1) Cross sectional (2) Animal study (3) Cell study (4) Cross sectional (5) Cross sectional (6) Animal study (7) Retrospective study	Pauci-immune glomerulonephritis	(1) 246 humans (2) 15 Wistar Kyoto rats (3) Human neutrophils (4) 11 humans (5) 15 humans (6) 10 Wistar Kyoto rats (7) 13 humans	Molecular mimicry with Gram negative bacteria

Roth et al. [21]	(1) Cross sectional (2) Animal study	ANCA glomerulonephritis	(1) 680 patients (2) 10 Wistar Kyoto rats	Molecular mimicry with Gram negative bacteria

Huugen et al. [22]	Animal study	Anti-MPO IgG-induced vasculitis	*Mpo* ^−/−^ mice and C57BL/6 mice	Bacterial lipopolysaccharide

Peschel et al. [23]	Cross sectional	ANCA glomerulonephritis	(1) 11 humans (2) 5 humans (controls)	Molecular mimicry with Gram negative bacteria

**Table 2:** Study characteristics of relevant studies in IgA vasculitis are shown. Influence of streptococcal proteins and ASO titers has been investigated most intensely. However, there might also be a role for *Staphylococcus aureus, Helicobacter pylori*, or *Haemophilus parainfluenzae *antigens in the pathogenesis of IgA vasculitis.

Reference	Design	Disease	Subjects	Biological agents
Masuda et al. [27]	Cross sectional	IgA vasculitis	33 patients	Group A streptococci and their antigen Nephritis associated plasmin receptor (NAPl-r)

Al-Sheyyab et al. [28]	Prospective case control	IgA vasculitis	48 children 48 controls	Antistreptolysin O titer

Schmitt et al. [29]	Cross sectional	IgA nephropathy/IgA vasculitis	16 humans (IgA nephropathy) 17 humans (IgA vasculitis)	IgA-binding M proteins of group A streptococci

Hirayama et al. [30]	Cross sectional	IgA vasculitis	6 patients 45 controls	*Staphylococcus aureus *

Li et al. [31]	Cross sectional	IgA vasculitis/membranous nephropathy/lupus nephritis	IgA vasculitis (*n* = 10), membranous nephropathy (*n* = 9), and lupus nephritis (*n* = 27)	*Helicobacter pylori *

Ogura et al. [32]	Cross sectional	IgA nephropathy/IgA vasculitis	32 children	*Haemophilus parainfluenzae *

**Table 3:** Study characteristics of relevant studies in IgA nephropathy are shown. Several bacterial strains have been identified by *in vivo* and *in vitro* experiments. There might be a pivotal role of Streptococci, *Helicobacter pylori, Haemophilus parainfluenzae, Haemophilus influenzae*, and *Staphylococcus aureus* in the disease onset or progression of IgA nephropathy as has been depicted.

Reference	Design	Disease	Subjects	Biological agents
Huang et al. [35]	Cross sectional	IgA nephropathy	106 patients	*Streptococcus *sp.,* Neisseria *sp.,* Haemophilus parainfluenzae, Staphylococcus *sp., *Bacillus proteus, *and*Streptococcus pneumoniae *

Nagasawa et al. [36]	Cohort study	IgA nephropathy	68 IgA nephropathy patients and 28 controls	*Haemophilus segnis, Campylobacter rectus, *and *Treponema *sp.

Drew et al. [41]	Cross sectional	IgA nephropathy	IgA nephropathy (35), systemic lupus erythematosus (6), membranous nephropathy (8), anti-GBM disease (6), and controls (20)	Pneumococcal polysaccharides

Schmitt et al. [29]	Cross sectional	IgA nephropathy IgA vasculitis	16 humans (IgA nephropathy) 17 humans (IgA vasculitis)	IgA-binding M proteins of group A streptococci

Schmitt el al. [39]	Cross sectional	IgA nephropathy	IgA nephropathy (21) Controls (83)	Streptococci

Nishikawa et al. [48]	Cell study	IgA nephropathy	Lymphocytes from IgA nephropathy patients	Streptococci

Chao et al. [56]	Animal study	IgA nephropathy	B-cell-deficient mice	Pneumococcal C-polysaccharide

Liu et al. [49]	Cross sectional	IgA nephropathy	27 patients	Haemolytic streptococcus and lipopolysaccharide

Kusano et al. [37]	Cross sectional	IgA nephropathy	32 IgA nephropathy patients 141 controls	*Helicobacter pylori*and*Actinomyces israelii *

Kusano et al. [38]	Cohort study	IgA nephropathy	55 patients	*Helicobacter pylori *

Yang et al. [50]	Cell study	IgA nephropathy	B cell line DAKIKI cells	*Helicobacter pylori *

Barratt et al. [42]	Cross sectional	IgA nephropathy	22 IgA nephropathy patients 9 controls	*Helicobacter pylori *

Suzuki et al. [40]	Cross-sectional	IgA nephropathy	44 IgA nephropathy patients 62 controls	*Haemophilus parainfluenzae *

Suzuki et al. [43]	Cross-sectional	IgA nephropathy	44 IgA nephropathy patients 62 controls	*Haemophilus parainfluenzae *

Suzuki et al. [51]	Cell study	IgA nephropathy	Tonsillar lymphocytes	*Haemophilus parainfluenzae *

Suzuki, et al. [52]	Cell study	IgA nephropathy	Tonsillar lymphocytes	*Haemophilus parainfluenzae *

Fujieda et al. [53]	Cell study	IgA nephropathy	Tonsillar mononuclear cells	*Haemophilus parainfluenzae *

Sunaga et al. [54]	Cell study	IgA nephropathy	Tonsillar mononuclear cells	*Haemophilus parainfluenzae *

Yamamoto et al. [57]	Animal study	IgA nephropathy	120 C3H/HeN mice	*Haemophilus parainfluenzae *

Nozawa et al. [55]	Cell study	IgA nephropathy	Tonsillar T cells	*Haemophilus parainfluenzae *

Ogawa et al. [44]	Cell study	IgA nephropathy	Glomerular IgA deposits	*Haemophilus influenzae *

Hirabayashi et al. [45]	Cross sectional	IgA nephropathy	24 IgA nephropathy patients 22 controls	*Escherichia coli *and* Haemophilus influenzae *

Shimizu et al. [46]	Cross sectional	IgA nephropathy, Post-MRSA glomerulonephritis	IgA nephropathy (*n* = 16) and post-MRSA infection GN (*n* = 19) Controls (*n* = 13)	*Staphylococcus aureus *

Koyama et al. [47]	Cross sectional	IgA nephropathy	Glomeruli from 238 kidney biopsies	*Staphylococcus aureus *

Sharmin et al. [58]	Animal study	IgA nephropathy	Balb/c mice (Th2 dominant type) and C57BL/6 mice (Th1 dominant type)	*Staphylococcus aureus *

Zhang et al. [59]	Animal study	IgA nephropathy	Balb/c mice	*Staphylococcus aureus *

**Table 4:** Study characteristics of relevant studies in nephrotic syndrome are shown. There are a small number of studies indicating a role of *Helicobacter pylori* infection in membranous nephropathy. Microbial agents in the other entities have not been studied yet. Moreover, conflicting results have been presented examining the role of infections prior to onset of nephrotic syndrome.

Reference	Design	Disease	Subjects	Biological agents
Moriyama et al. [68]	Cross sectional	Membranous nephropathy	32 patients	*Helicobacter pylori *

Li et al. [31]	Cross sectional	IgA vasculitis/membranous nephropathy/lupus nephritis	IgA vasculitis (*n* = 10), membranous nephropathy (*n* = 9), and lupus nephritis (*n* = 27)	*Helicobacter pylori *

Nagashima et al. [69]	Cross sectional	membranous nephropathy	16 patients	*Helicobacter pylori *

Gulati et al. [70]	Retrospective study	Primary nephrotic syndrome	37 children	Non-*Escherichia coli* organisms, urinary tract infection

Adedoyin et al. [71]	Cross sectional	Primary nephrotic syndrome and acute glomerulonephritis	67 children	Coliforms, *Klebsiella *sp., and *Staphylococcus aureus*, urinary tract infection

**Table 5:** Study characteristics of relevant studies in systemic lupus erythematosus and lupus nephritis are shown. Most experience is gathered from mouse models with induction of lupus-specific antibodies after injection of lipopolysaccharide. Lipopolysaccharide along with specific bacterial strains was capable of worsening kidney involvement in lupus mouse models, indicating a potential role of bacterial antigens in the progression of lupus nephritis.

Reference	Design	Disease	Subjects	Biological agents
Kowal et al. [75]	Cell study	*Lupus nephritis *	1 patient	Anti-bacterial antibodies which bind double-stranded DNA

Chowdhry et al. [76]	Cell study	*Lupus nephritis *	1 patient	Antibodies binding bacterial polysaccharide and glomeruli

Gilkeson et al. [79]	Animal study	*Lupus nephritis *	BALB/c and C57BL/6 mice	*Escherichia coli* dsDNA

Izui et al. [80]	Animal study	*SLE *	Mice	Lipopolysaccharides

Fournié et al. [81]	Animal study	*SLE *	Athymic C57BL/6 nude mice	Lipopolysaccharide

Granholm and Cavallo [82]	Animal study	*Lupus nephritis *	BXSB mouse	Lipopolysaccharide

Granholm and Cavallo [83]	Animal study	*Lupus nephritis *	NZB/W mice	Lipopolysaccharide

Cavallo and Granholm [84]	Animal study	*Lupus nephritis *	MRL/lpr mice	Lipopolysaccharide

Anders et al. [77]	Animal study	*Lupus nephritis *	MRL/lpr mice	*Escherichia coli *

Deng and Tsokos [78]	Animal study	*SLE *	MRL/*lpr*/*2J* mice, F_1_ (NZB/W F_1_) mice, MRL/MpJ mice, B6.MRL/*lpr* mice, CD40 ligand knockout mice, and C57BL/6 (B6) mice	Cholera toxin B
